# Internet as a Source of Long-Term and Real-Time Professional, Psychological, and Nutritional Treatment: A Qualitative Case Study Among Former Israeli Soviet Union Immigrants

**DOI:** 10.2196/jmir.7130

**Published:** 2017-02-03

**Authors:** Anat Gesser-Edelsburg, Svetlana Shalayeva

**Affiliations:** ^1^ University of Haifa Health and Risk Communication Research Center School of Public Health University of Haifa Haifa Israel; ^2^ School of Public Health University of Haifa Haifa Israel

**Keywords:** long-term care, real-time systems, online systems, health psychology, nutrition therapy, qualitative research, former Soviet Union immigrants

## Abstract

**Background:**

The Internet is considered to be an effective source of health information and consultation for immigrants. Nutritional interventions for immigrants have become increasingly common over the past few decades. However, each population of immigrants has specific needs. Understanding the factors influencing the success of nutrition programs among immigrants requires an examination of their attitudes and perceptions, as well as their cultural values.

**Objective:**

The purpose of this study was to examine perceptions of the Internet as a tool for long-term and “real-time” professional, psychological, and nutritional treatment for immigrants from the former Soviet Union who immigrated to Israel (IIFSU) from 1990 to 2012.

**Methods:**

A sample of nutrition forum users (n=18) was interviewed and comments of 80 users were analyzed qualitatively in accordance with the grounded theory principles.

**Results:**

The results show that IIFSU perceive the Internet as a platform for long-term and “real-time” dietary treatment and not just as an informative tool. IIFSU report benefits of online psychological support with professional dietary treatment. They attribute importance to cultural customization, which helps reduce barriers to intervention.

**Conclusions:**

In light of the results, when formulating nutritional programs, it is essential to have a specific understanding of immigrants’ cultural characteristics and their patterns of Internet use concerning dietary care.

## Introduction

### Internet as a Source of Consultation and Support for Immigrants

Personalized treatment with psychological support in nutritional intervention over the Internet increases the effectiveness of interventions [1,2]. Social media offer a platform for long-term and real-time support with professional consultation, rather than frontal meetings [3-5].

The Internet is considered one of the most effective tools for reducing medical gaps between immigrants and locals [6,7]. It is an important tool for overcoming immigrants’ feelings of estrangement and language barriers [8]. Research in different countries has shown that, compared with the majority population, immigrants generally have less access to health services and health information, mainly due to language and cultural barriers [9-11]. Subsequently, online health services offered by multicultural professionals serve as a tool for reducing communication barriers, thereby improving the quality and delivery of health services [12].

The use of Internet among immigrants is correlated with online health literacy. However, there are populations of immigrants, like Russian immigrants, who are well educated, but whose health literacy is low, and consequently, their knowledge of health and nutrition is poor [13,14]. This may present a challenge in a new country with a different culture and unfamiliar eating habits. Therefore, it is essential to mentor immigrants while considering their cultural needs.

### Internet Intervention Programs and Real-Time Support

Continued use of nutrition interventions by health professionals is associated with successful results among patients [2,15]. Also, real-time support is essential to the success of the intervention [16].

Despite the benefits of the Internet as a platform for dietary treatment including professional and psychological support, few studies have focused on long-term and real-time online nutritional intervention. An interdisciplinary review [2] has shown that most of the studies in the field are randomized controlled trials with a maximum period of 13 months per intervention. Qualitative studies that examined attitudes of participants in nutritional interventions often included semistructured and indepth interviews [17,18] and focus groups [18].

Studies that were conducted among immigrants involved school children, adolescents, and young adults and their families [19]; overweight and obese and sick adults [20]; older adults [17]; pregnant women [21]; or males and females [22]. Some of the studies were conducted on bilingual health assistants [21,22]. Nearly all the studies considered first-generation immigrants and were small scale.

These interventions usually included a predefined plan prepared by the authors [23-25]. The participants did not always have the opportunity to ask questions or to obtain information about personal issues in real time. Long-term online nutritional treatment for Russian immigrant populations has not been studied. This study examines the use of Internet as a tool for long-term dietary counseling and psychological support for immigrants from the former Soviet Union in accordance with their cultural needs.

### Characteristics of Russian Immigrants in Israel

IIFSU tend to have advanced degrees [26], but this is not always reflected in health literacy. Despite their high level of education, their nutritional knowledge and dietary intake are poor.

After immigration, IIFSU often see themselves as belonging to both the Russian and Israeli cultures, meaning they have 2 cultural identities [17]. After arriving in Israel, nutrition becomes more significant to them [13,14]. In Israel, eating habits are different from that in Russia. According to Lissak [27] and Biederman [28], in contrast with Soviet immigrants who arrived in Israel in the 1970s and sought to assimilate into the collective culture, the IIFSU showed a tendency toward acculturation [29] (eg, by opening Russian supermarkets, theaters, and bookstores). When immigrants rely on their originary ethnic group that speaks their language and abide by its customs and norms, including eating habits, such behavior increases their sense of security and facilitates gradual entry into the broader society [30-33]. Ben-Sira [34] argues that the IIFSU tend to maintain a diet high in fat and cholesterol based on eating habits from their country of origin. Despite being aware that such a diet can lead to obesity and associated health risks, only 40% of the IIFSU tend to choose healthy food. These eating habits of diets based mostly on bread and processed meats, along with other unhealthy lifestyles such as heavy smoking, high alcohol intake, and infrequent doctor visits for preventative care are all part of the life history and health experiences prior to the dissolution of the Soviet Union [35-40]. The first Israeli National Health and Nutrition Survey [41] showed that obesity was more prevalent among Soviet immigrants who arrived in the 1990s than among other ethnic groups in Israel. This study examines attitudes and perceptions toward the Internet as a tool for long-term personalized nutritional treatment and support for IIFSU. In this study, the nutrition counseling was provided by professional certified nutritionists whose public record of their credentials was presented either on a website, on their forum, or on a business webpage.

### Objectives

The objective of this study was to examine perceptions and attitudes about the use of Internet as a tool for dietary interventions among immigrants from the IIFSU. Specific objectives were (1) to examine the use of Internet as a source of long-term and real-time dietary treatment; (2) to examine benefits of online professional dietary treatment with psychological support among immigrants; and (3) to examine how the cultural customization helps reduce resistance and barriers to compliance with dietary treatment.

## Methods

### Study Design and Analysis

The study combined semistructured, indepth interviews, and comments made by Russian forum users on social networks (Facebook, odnoklassniki, vkontakte).

### Data Collection

All interviewees provided information about background variables including age, education, marital status and employment, country of origin, and year of immigration. Questions pertaining to study objectives covered the following issues: Internet as a source of information for health issues, Internet use by IIFSU, centrality in their lives, and lack of adherence in general and regarding nutritional care. The protocol included questions on interviewees’ perceptions on nutritional issues, about the Internet as a source of knowledge, and their experiences of nutritional therapy interventions via the Internet; their barriers and dilemmas concerning nutritional therapy, influenced by cultural characteristics, online health literacy, and acclimatization in the country. Participants were asked to compare face-to-face and Internet-based nutritional therapy.

### Summary of the Recruitment Population Study

Different subtypes of the IIFSU Internet users were selected: diverse ages, gender, socioeconomic status, and number of years in Israel (Table 1).

Recruitment was carried out through an open forum for nutritional counseling on social networks. All users received nutritional consultation and long-term treatment. Some were followed up from 1 month to several years. Eighteen indepth face-to-face interviews (between 40 and 60 min) were conducted at a time and place convenient to interviewees. Interviews were recorded and transcribed. Eighty users' comments from forums were analyzed using content analysis.

IIFSU, men and women aged 24-58 years, who immigrated to Israel from 1990 to 2012, most (14 out of 18) were found to hold BA degrees or higher and their socioeconomic statuses vary.

**Table 1:** Demographic data.

Interviewee	Gender	Age in years	Employment	Year of immigration	Years living in Israel	Age of immigration	Education
1	M	33	Hi-tech marketing manager	1993	22	12	BA
2	M	29	Computer science student	1999	16	14	BA student
3	M	40	Hi-tech engineer	1994	21	20	BA, MA student
4	F	25	Bookkeeper	1998	17	9	Vocational training
5	F	24	Dental assistant	2000	15	9	Vocational training
6	F	30	Hi-tech programmer	1991	24	6	BA
7	F	27	Social worker	1992	23	5	BA
8	F	53	Musician	1997	18	35	BA
9	F	40	Photographer	1998	17	24	Vocational training
10	F	31	Housewife	2012	3	29	BA
11	F	47	Saleswoman	2002	13	37	BA
12	M	56	Sports store manager	1990	25	31	MA
13	M	26	Construction worker	2000	15	11	High school
14	F	25	Architecture student	2003	12	13	BA student
15	M	58	Unemployed	1990	25	33	BA
16	F	55	Nurse	1998	17	38	BA
17	F	52	Factory worker	1991	24	28	BA
18	M	38	Lawyer	1993	22	16	BA

### Data Analysis

The audiotape was transcribed as soon as possible after each interview. Transcripts were then checked against the recordings to ensure accuracy. We conducted a content analysis of additional 80 users’ comments on nutritional forums. The analysis was conducted throughout the data collection process and the focus was on issues related to the research questions [42] and on themes that arose in the personal interviews.

The themes of the generic subjects that appeared in the content analyses of the users’ comments and the semistructured protocols were analyzed. Interviews and users’ comments were analyzed individually to identify key themes and subthemes. Data aggregation indicated a saturation point because there were repetitions of themes in the interviews and there was no need for more interviews [43]. The findings presented in the study are an integration of the forum users’ comments and the issues that emerged during the interviews.

### Validity and Reliability

We used the triangulation method including diverse data sources (tools). This approach uses cross-referencing data and validation. Use of multiple sources allows improved understanding, control, validity, and reliability of the findings. The study included personal interviews (semistructured protocol) and analysis of users’ comments from Internet forums. For validation, the results were compared with findings in the literature. The comparison showed similar conclusions. Consequently, the study received a basis for its validity [44]. In addition, recognition of the limitations helped improve the quality and validity of the study [45]. At each stage, the researcher compared and brought into line the participants’ views and the construction of those views by the researchers. To reinforce study reliability and credibility, different subtypes IIFSU were selected.

### Ethical Considerations

Application was submitted to the Faculty of Social Welfare and Health Sciences Ethics Committee for research with human subjects at Haifa University and full ethical approval (no. 106/14) was granted.

## Results

### Main Subthemes

Over the course of the research, common themes arose for different subpopulations of IIFSU, along with a distinct variance between those researched. We divided the results into 3 subthemes that are as follows: (1) receiving long-term treatment and real-time dietary consultation; (2) professional dietary treatment with psychological support; and (3) cultural tailoring to the needs of IIFSU.

### Receiving Long-Term Treatment and Real-Time Dietary Consultation

Most interviewees and most users’ comments indicate that the Internet offers an opportunity to receive long-lasting dietary treatment. Also, there is the option of real-time consultation from professionals. Nutrition forums serve as a therapeutic tool as discussed in the following.

#### Long-Term Therapist-Patient–Relationship Through Online Counseling

In Israel, professional certified independent nutritionists provide online nutrition counseling. They promote their online services mainly through social media, and provide them through online video chat, Facebook, or their forum. Online nutrition counseling is not regulated by law. Eleven of 18 interviewees (7 women, 4 men) sought long-term dietary counseling, an unlimited number of consultations like in other online programs or in the traditional clinics. Patients state their need for customized therapeutic framework for effective changing of behavioral habits. It is a long-term process and this is why they need long-term follow-up and encouragement.

With the professional counseling I receive on the Internet, I feel they can mentor me and help me to change my eating habits step by step. This way I achieve my goals. It helps me to learn about new culture and food. This difficult process of changing habits takes time…So it is important to me to have follow-up and discipline, since I progress better like this… It is easier for me to deal with the problems with a therapist than alone...

M, 29

#### Real-Time Connection

Receiving counseling when difficulties or questions arise, which are not predetermined enables the patient to follow through on the therapist’s recommendations. Twelve of 18 interviewees (8 women and 4 men, 25-35 years, with no difference in education or duration of residence in Israel), positively noted the option of a real-time relationship with their consultant via the Internet:

I keep having questions all the time, which I want answers to, and through the Web I can ask those questions immediately in real time. I want to ask the dietitian’s opinion in real time, go over my experiences together, exchange experiences, write down what I’m going through,...I think that doing this in a clinical setting is problematic, since there is an accessibility issue and you have to reschedule appointments. Online I can address professionals any time and talk with them, which is impossible to do at the clinic.

M, 33

### Professional Dietary Treatment With Psychological Support

#### Internet as a Place for Consulting Professionals

Thirteen of 18 interviewees (men and women, 24-58 years) regard the Internet as a venue for receiving dietary treatment and consultation, instead of frontal consultations with dietitians. Moreover, the saturation of dietary information on the Web is overwhelming and requires guidance. Russian users reveal that they only trust professionals. Users’ comments revealed that they sought an authoritative source to assist in decision making. The study participants reported that they were looking for guidance from a licensed professional. Before they began the counseling, they reported that they checked the counselor’s details on Google or that they asked to see professional certification to validate whatever licensing and degrees they claimed to have. According to the participants, a good nutritionist was one with professional training.

I improve my diet based on the professional consultation I get in forums. There are too many information and blogs about nutrition, but not many good professionals. It's critical for me to get a proper consultation which will influence my food intake.

M, 29

#### Internet as a Source of Psychological Support During the Treatment

Apart from the professional consultation, patients express a need for encouragement and motivation. Also mentoring and coaching helps change habits. Eight of 11 women stated they had used the Internet in situations of uncertainty. Through the connection which enables sharing difficulties and getting psychological support, the experience of treatment becomes more effective. The patient senses the therapists’ support, when he needs their guidance, as one of the interviewees notes this:

To me, changing eating habits is like changing culture. It’s changing your roots. It seems almost impossible, because my eating culture is a part of me…but I do it because it is important for me to improve my health, and it’s important for me physically. From a psychological standpoint, it is very difficult. It takes a lot of time and professional support who can encourage, explain, and give us the appropriate tools to deal with temptation and pressure.

M, 29

### Cultural Tailoring to the Needs of IIFSU

Being immigrants influences the compliance with dietary treatment for several reasons:

#### Language

Eleven of 18 interviewees stated that speaking Russian is important in dietary treatment. Those who chose to receive counseling in Russian stressed that it was easier as they conduct their personal lives in Russian. They see themselves as part of a Russian community in Israel.

I prefer someone who speaks Russian, but who lives in Israel, someone like me… So that we will have a better understanding…Although I am fine with Hebrew, it is better speaking with someone who knows your native language to make matters clearer, it makes it easier for me.

F, 27

Segmentation of the findings regarding language choice for treatment was observed among users from different age groups. Both the younger (aged 20-40) and older (aged 40-56) users noted a preference for counseling in Russian. A difference of opinion was also observed among those who immigrated to Israel as children (ages 2-14), as well as among those who immigrated after age 15, and had been living in Israel between 1 and 10 years. They all shared a common characteristic: patients whose daily routines were in Russian preferred a Russian-speaking therapist. That means the phenomenon has less to do with the age and amount of time since immigrating, and more to do with the role of Russian in their daily lives.

#### Mentality and Culture During Treatment

Nine of 18 patients expressed the need for a therapist who was also a Russian immigrant. This fostered mutual understanding and a sense of identification. Furthermore, it helps cope with difficulties in a new culture.

I prefer that the therapist be Russian...Not only speaking the language, but also knowing what Russian culture is, what a Russian upbringing is...I think it’s the mentality, I mean that they will be someone like me who lives in Israel and knows the Russian mentality. That is why I think that a Russian dietitian understands me better. When I say a Russian lifestyle with a Russian mentality – it is because I speak the Russian language, eat Russian foods and take part in Russian culture and understanding. It’s hard to explain, but it’s like dating an Israeli. It’s fun and nice but we’re not on the same wavelength, we don’t fully understand each other.

F, 30

## Discussion

### Principal Findings

IIFSU look for professional consultation with psychological support that takes into account their cultural identity. The IIFSU interviewed tend to be more educated than the general population. This might explain the extensive use of the Internet as an informative and therapeutic instrument. However, these data contradict findings in the literature, in which immigrant populations of disadvantaged groups (elderly, poor, and chronically ill), use the Internet less, and do so less rationally [15,46,47]. All the users in this study stated that the Internet was effective, empowering, and helpful for dietary issues.

Despite their high level of education, a gap in health literacy remains [48]. The population interviewed tends to have unhealthy eating habits, reflecting the eating habits in their native country and the complexities of immigration. The study participants reported on their eating habits to the interviewer. The reports expose eating habits that include a diet high in fat, sweets, and juices, with little consumption of vegetables and water. The Russian interviewees sought dietary support in terms of both health and culture. This is in keeping with the importance placed on “cultural sensitivity” in the literature. Cultural sensitivity entails awareness of cultural similarity and difference, which influence worldviews, values and beliefs, learning processes, and behavior [49]. Differences in culture must therefore be considered at every stage in dietary intervention. This may guarantee the efficacy of the treatment offered to Internet users [50].

According to the literature, publishing dietary and medical content in the immigrants’ language and making it culturally appropriate helps carry messages of health [15,46]. Most users in this study claimed that speaking Russian was a crucial factor in creating trust. However, it appears that a preference for Russian as a language for dietary treatment depends on the parameters. This study is the first to assess the differences among the Russian immigrants who prefer Russian for their online treatment. The variance in the distribution of preferences did not depend on age group or number of years since immigrating; rather, those whose daily routines were in Russian preferred a Russian-speaking therapist.

The Russian interviewees stressed the importance of the therapist’s mentality and knowledge of both Russian and Israeli cultures. They need to be counseled by a Russian immigrant to Israel. The professional literature supports these findings. One of the measures for diminishing health literacy gaps between the 2 population strata such as Russian immigrants and the permanent residents of Israel is the communication that stems from the patient’s dominant culture [51]. Online health services offered by multicultural professionals are a valuable tool for reducing communication barriers [12] and can increase the compliance with dietary treatment. This may be why an online therapist can replace frontal treatment at a clinic.

In this study, participants stressed that they preferred treatment on the Internet because it enabled the creation of a long-term relationship between the therapist and the patient. Furthermore, long-term online dietary intervention allows for a real-time relationship and facilitates compliance. Thus, the patient could receive continual reinforcement and maintain motivation.

Most studies conducted on intervention programs were limited by time, where professional counseling was offered at predetermined points at the beginning, middle, and end [23,25,52]. Based on our findings, users valued counseling during crisis and periods of success, which are not predetermined.

Russian immigrants in the study turned to a dietary treatment which includes not only professional consultation, but also psychological support. Studies show that Internet users look for nutritional information from evidence-based and easily recognizable sources [53]. In addition, users need psychological support and interventional guidance [54,55]. Providing professional consultation with coaching methods and psychological support show high compliance and usability among the IIFSU in this study.

In this study, this finding was particularly notable among young users (aged 25-35 years), regardless of difference in education or time spent in Israel, with a majority of women over men. In the professional literature, several explanations emerged for our study’s findings. A Russian study [56], which performed an extensive examination of the behavioral patterns of more than 16 million users of the popular Russian social network (My.Mail.Ru), discovered that women were more active on the Internet than men. In addition, women were willing to disclose more details about themselves and share personal information, such as their physical appearance, hobbies, occupation, family status, and more. Similar findings have been noted among social network users around the world [57,58].

Creating professional and emotional support during the intervention that considers the cultural needs of patients fosters higher health care quality in cross-cultural situations [59].

### Limitations

The limitations of this study are that as it is a qualitative study, it does not include a representative sample of the study population. In addition, the next step should be further validation through randomized controlled trials and implementation.

In our study, we did not compare the efficacy of the nutritional counseling online versus frontal nutritional counseling, as our focus was on the perceptions of the participants regarding the Internet as a tool for long-term and “real-time” professional, psychological, and nutritional treatment. Over the course of the study, some of the participants reported weight loss and an improvement in clinical indicators. However, as noted, we did not gather quantitative clinical data that would enable a comparison with one-on-one nutritional counseling. Notwithstanding this, in the literature there are studies that indicate positive results when using online nutritional counseling, which have been found to lead to a significant improvement in nutrition and to a healthier lifestyle [60], weight loss, rise in consumption of fruits and vegetables, a decrease in consumption rate of fats and sugar, and a decrease in calorie consumption [54]. Other studies did not find a significant difference in BMI and the BMI z-score between the experimental group and the control group [61], nor did they find significant differences in serum, blood pressure, anthropometry, social support, and cholesterol [62].

Given that the field of online nutritional counseling is still a relatively new field, we think that more studies are needed in order to assess the effectiveness of online counseling as compared with on-one-one nutritional counseling. We can also assume that in the future, technological advances will improve the efficacy of online counseling, whether by incorporating virtual smart agents, and augmented and virtual reality, or by an increased presence of peers who will empower the patients, leading to more effective counseling.

### Conclusions

This study is an examination of the perceptions of IIFSU regarding the Internet as a tool of nutritional treatment. It included long-term dietary treatment, real-time counseling, and nutritional information with psychological support. All the interventions were carried out with the immigrants’ cultural needs in mind. Our study is the first to examine unique cultural perceptions and beliefs, which affect the rate of response to dietary treatment among a minority population.
